# Human B Cell-Derived Lymphoblastoid Cell Lines Constitutively Produce Fas Ligand and Secrete MHCII^+^FasL^+^ Killer Exosomes

**DOI:** 10.3389/fimmu.2014.00144

**Published:** 2014-04-02

**Authors:** Matthew W. Klinker, Vincent Lizzio, Tamra J. Reed, David A. Fox, Steven K. Lundy

**Affiliations:** ^1^Graduate Program in Immunology, University of Michigan, Ann Arbor, MI, USA; ^2^Division of Rheumatology, Department of Internal Medicine, University of Michigan, Ann Arbor, MI, USA

**Keywords:** lethal exosomes, transplant tolerance, Epstein–Barr virus, microvesicles, T cell apoptosis, regulatory B cells, killer B cells

## Abstract

Immune suppression mediated by exosomes is an emerging concept with potentially immense utility for immunotherapy in a variety of inflammatory contexts, including allogeneic transplantation. Exosomes containing the apoptosis-inducing molecule Fas ligand (FasL) have demonstrated efficacy in inhibiting antigen-specific immune responses upon adoptive transfer in animal models. We report here that a very high frequency of human B cell-derived lymphoblastoid cell lines (LCL) constitutively produce MHCII^+^FasL^+^ exosomes that can induce apoptosis in CD4^+^ T cells. All LCL tested for this study (>20 independent cell lines) showed robust expression of FasL, but had no detectable FasL on the cell surface. Given this intracellular sequestration, we hypothesized that FasL in LCL was retained in the secretory lysosome and secreted via exosomes. Indeed, we found both MHCII and FasL proteins present in LCL-derived exosomes, and using a bead-based exosome capture assay demonstrated the presence of MHCII^+^FasL^+^ exosomes among those secreted by LCL. Using two independent experimental approaches, we demonstrated that LCL-derived exosomes were capable of inducing antigen-specific apoptosis in autologous CD4^+^ T cells. These results suggest that LCL-derived exosomes may present a realistic source of immunosuppressive exosomes that could reduce or eliminate T cell-mediated responses against donor-derived antigens in transplant recipients.

## Introduction

Allograft rejection mediated by immune responses to donor-derived antigens remains a significant concern following organ transplantation (1). Alloreactive T cells are thought to be central to the process of rejection, and most recipients of organ transplants receive long-term treatment with immunosuppressive drugs that globally suppress T cell responses. The broad immunosuppression mediated by these drugs can lead to increased susceptibility to infection and reduced cancer surveillance in patients, and therefore a therapeutic means of targeting alloantigen-specific T cells in transplant recipients would be a profound advancement over current treatments.

Exosomes are extracellular vesicles approximately 50–90 nm in diameter that are secreted by a variety of immune cells. In antigen-presenting cells (APC), exosomes originate from the same intracellular compartment where newly synthesized and recycled MHCII molecules are loaded with peptides derived from endocytosed proteins (2). While most reports suggest that the majority of exosomes released by APC activate T cells, immunosuppressive APC-derived exosomes have been described as well. Stimulation of murine bone marrow-derived dendritic cells (BMDC) with IL-10 resulted in the production of exosomes capable of suppressing an immune response *in vivo* (3). Additionally, BMDC transfected with a vector expressing the gene encoding the apoptosis-inducing molecule Fas ligand (FasL) produced MHCII^+^FasL^+^ exosomes that were able to suppress an immune response *in vivo* (4). Importantly, the suppression mediated by the MHCII^+^FasL^+^ exosomes was antigen-specific and FasL-dependent. Naturally occurring MHCII^+^FasL^+^ exosomes have been identified as well, and these endogenously produced exosomes demonstrated antigen-specific immune suppression upon transfer to recipient mice (5). Immunosuppressive exosomes also were effective in prolonging graft survival in a cardiac allograft model in rats (6). For the suppression of human immune responses, exosomes may represent a safer alternative to regulatory cells for immunotherapy because the phenotype of exosomes is static, whereas regulatory cells can potentially differentiate into effector cells after transfer (7). Therefore, a cost-effective and reliable method for producing immunosuppressive MHCII^+^FasL^+^ exosomes is potentially of great value for the development of exosome-based immunotherapies.

While FasL is most frequently studied in T cells or natural killer (NK) cells, FasL expression by B cells has been reported in numerous conditions (8). B cells expressing FasL were initially observed following stimulation of murine B cells with mitogens (9). Some forms of B cell-derived cancers in humans have been reported to express FasL, including multiple myeloma, B cell chronic lymphocytic leukemia, and large B cell lymphoma (10–12). FasL-expressing B cells were induced by infection with the parasitic worm *Schistosoma mansoni* in mice, and their increased frequency coincided with greater levels of apoptosis in CD4^+^ T cells (13). There is also evidence that FasL-expressing B cells may play a role in the regulation of autoimmunity and maintaining self-tolerance. Activated B cells expressing FasL and TGFβ have been reported to delay the onset of diabetes in non-obese diabetic (NOD) mice, and the frequency of FasL^+^ B cells is reduced in mice with severe autoimmune arthritis relative to those with mild or no arthritis (14, 15). Mice with a B cell-specific loss of FasL spontaneously develop autoantibodies despite the fact that T cells in these animals are FasL-sufficient, demonstrating that B cell expression of FasL plays a role in maintaining immune homeostasis (16). Bone marrow cells treated with the TLR-9 agonist CpG are enriched for B cells that express high levels of FasL and protect NOD mice from type 1 diabetes upon adoptive transfer (17). B cells from Fas-deficient MRL/lpr mice also express high levels of FasL, and kill Fas-susceptible target cells with an efficiency similar to that of NK cells (18). In a male-to-female transplantation model, transfer of B cells from wild-type males prior to skin grafting induced tolerance to H–Y antigen in female recipients, whereas FasL-deficient B cells were unable to transfer tolerance (19). Taken together, these studies demonstrate that FasL production by B cells is potentially important for suppressing immune responses in many settings, including tolerance of allografts.

In the current study, we report that a high frequency of lymphoblastoid cell lines (LCL) derived from human peripheral blood B cells constitutively produce FasL protein. Importantly, all LCL-tested secreted MHCII^+^FasL^+^ exosomes, and using two independent experimental approaches, we demonstrated that LCL-derived exosomes can induce targeted apoptosis in activated CD4^+^ T cells. Therefore, we propose that exosomes produced by a donor-derived LCL may represent a reliable source of alloantigen-specific immunosuppressive exosomes that could potentially be used to tolerize transplant recipients.

## Materials and Methods

### Preparation of peripheral blood mononuclear cells

All donors provided informed consent prior to their participation in this study. Blood was obtained by venipuncture and collected into syringes containing sodium heparin. Following a 1:1 dilution with un-supplemented RPMI 1640, blood was gently layered onto Histopaque-1077 (Sigma-Aldrich) in 50 mL centrifuge tubes. Buffy coats containing peripheral blood mononuclear cells (PBMCs) were collected from tubes following centrifugation at 1,200 × *g* for 30 min at 20°C.

### Cell lines

Lymphoblastoid cell lines were produced according to established techniques for the transformation of B cells by Epstein–Barr virus (EBV) using the non-replicating laboratory strain B95-8 (American Type Culture Collection) (20). Cell lines used were derived from either healthy donors and generated in our laboratory, or were from a collection of LCL derived from monozygotic twin pairs discordant for rheumatoid arthritis (a kind gift from Dr. Joseph Holoshitz, University of Michigan) (21). LCL were maintained in RPMI 1640 media supplemented with 20% FBS, 2% l-glutamine, 1% penicillin/streptomycin, 1% non-essential amino acids, and 1% sodium pyruvate. Most cell lines were kept in culture continuously for longer than 2 months with no detectable changes in growth, viability, or experimental results. Once or twice per week, LCL cultures were split 1:3, and kept in a 37°C, 5% CO_2_ incubator.

### Exosome isolation and preparation from LCL culture supernatants

Exosome-free FBS was produced by centrifuging FBS overnight at 100,000 × *g* to remove any bovine-derived exosomes. Culture supernatants from LCL cultures were spun at 500 × *g* for 10 min to remove cells, followed by a spin at 10,000 × *g* for at least 1 h to remove large cellular debris and microparticles. Exosomes were then obtained by centrifugation at 100,000 × *g* for 1–4 h. The resulting exosome pellets were diluted once with PBS prior to another 100,000 × *g* centrifugation, after which pellets were re-suspended in a small volume of PBS. Protein concentration was used as a proxy measure for the amount of exosomes in a given re-suspension, and was determined by BCA assay. The presence of microparticles with sizes that were consistent with exosomes was confirmed by transmission electron microscopy (data not shown). In some cases, supernatant from bulk cultures of LCL were concentrated using centrifuge tubes equipped with a 100-kDa filter prior to exosome isolation by ultracentrifugation.

### Immunoblotting

Lymphoblastoid cell lines and LCL-derived exosomes were lysed in SDS buffer prior to separation by SDS-PAGE and transfer to a PVDF membrane. Membranes were blocked using manufacturer recommended buffers specific to each antibody and incubated with polyclonal rabbit anti-FasL IgG (Cell Signaling), mouse anti-HLA-DR (Abcam, clone TAL 14.1), or polyclonal rabbit anti-β-Actin (Cell Signaling). Antibody binding was detected with an anti-rabbit or anti-mouse IgG–HRP secondary antibody (Cell Signaling) and ECL reagent (Thermo Scientific).

### Flow cytometry

PE-conjugated anti-FasL and isotype control antibody were obtained from Biolegend (clone NOK-1). LCL were incubated with anti-CD16/CD32 Fc Block (BD Biosciences) prior to staining and analyzed on a Beckman Coulter FC500 flow cytometer. For intracellular staining, LCL were fixed for 20 min at room temperature in 4% PFA, washed three times with PBS, and permeabilized with 0.5% saponin buffer prior to staining with anti-FasL. For apoptosis staining, annexin-V-FITC (eBioscience) was used to identify cells in early apoptosis and propidium iodide was used to identify dead cells. Data were analyzed using Cytobank web-based software (22) or FlowJo v7.6.5 (Tree Star, Inc.).

### Density gradient centrifugation

Serial dilutions of iodixanol (OptiPrep; Sigma-Aldrich) were prepared with PBS, with densities ranging from 1.03 to 1.27 g/mL. One milliliter of each density fraction was added sequentially to an ultracentrifuge tube so as to maintain a discontinuous gradient. A sample of LCL-derived exosomes in solution was placed on top of the density gradient and centrifuged at 100,000 × *g* for 1 h. Layers were then removed to separate tubes and diluted in PBS, and diluted fractions were centrifuged in individual tubes overnight at 100,000 × *g*. Pellets from each density fraction were lysed in an equal amount of SDS buffer, and interrogated for FasL and MHCII by immunoblot.

### Exosome bead capture experiments

Polystyrene beads (~6.7 μm diameter) coated with streptavidin were obtained from Corpuscular Inc. or Spherotech Inc. Beads were coated for 1 h at 20°C with biotinylated antibodies against human HLA-DR (Biolegend, clone L243), or the appropriate isotype control antibody. After washing, antibody-coated beads were incubated for 2–3 h with ultracentrifuge-purified exosomes at 4°C with gentle agitation. Unbound exosomes were then washed away, and bead-bound exosomes were stained for FasL and subsequently analyzed on a Beckman Coulter FC500 flow cytometer.

### Exosome-induced apoptosis assays (TT peptide)

Peripheral blood mononuclear cells were isolated from whole blood and stimulated for 12 days with an immunodominant peptide of tetanus toxoid (TT) (10 μg/mL). This donor had received a scheduled booster vaccination against tetanus within 2 months of these experiments. CD4^+^ T cells were separated from PBMCs cultures by negative selection by MACS (Miltenyi Biotec) and cultured overnight with exosomes (156 μg total protein/mL for the experiment shown) derived from an autologous LCL in the presence or absence of the stimulating peptide. The number of exosomes used for each experiment was optimized based on the yield from ultracentrifugation and was the same for all wells of the experiment. Total exosome protein content measurement was used to assess the inter-experimental variability and ranged from 100 to 250 μg/mL in all experiments. The activity of FasL was blocked in culture by the addition of 10 μg/mL of anti-FasL antibody (BioLegend; clone NOK-1). Apoptosis was assessed in T cells by annexin-V/propidium iodide staining among activated (CD4^+^CD62L^neg^) T cells.

### Exosome-induced apoptosis assays (SEA)

CD4^+^ T cells were isolated from whole blood by RosetteSep Human CD4^+^ T cell enrichment cocktail (Stem Cell Technologies) and stimulated for 6 days with Staphylococcal enterotoxin A (10 ng/mL; Sigma-Aldrich) in the presence or absence of exosomes (127 μg/mL) purified from an autologous LCL. T cells were then harvested and apoptosis was assessed by annexin-V/propidium iodide staining among total CD4^+^ T cells.

## Results

### High frequency of LCL constitutively produces intracellular FasL protein

**Figure 1:**
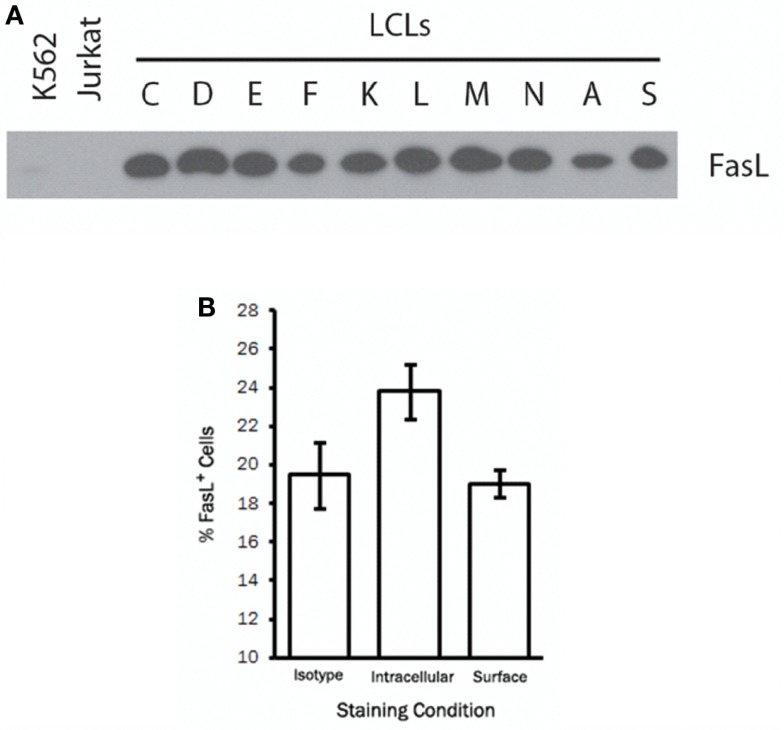
**A high frequency of LCL constitutively produce intracellular FasL protein**. **(A)** Equivalent numbers of LCL and control cell lines were lysed and probed for expression of FasL protein by immunoblot. **(B)** LCL were fixed and stained for surface FasL, or permeabilized with saponin buffer for intracellular FasL staining by flow cytometry. An isotype control antibody was used to control for non-specific binding on the cell surface and following cell permeabilization. Results are representative of at least three LCL tested.

To study the regulation and trafficking of FasL in B cells, we obtained several B cell-derived cell lines to screen for FasL expression. Among the cell lines tested were several LCL generated by transformation of human peripheral blood B cells with the attenuated B95-8 clone of EBV (23). By immunoblotting for FasL, we found that all LCL tested (>20 independent lines) displayed robust and constitutive expression of FasL protein (Figure 1A). Expression of FasL in the myeloid leukemia cell line, K562, and the T cell leukemia line, Jurkat, was not detected. Other B cell-derived cell lines such as human B cell lymphomas were only sporadically FasL^+^ (data not shown). We therefore conclude that a high frequency of cell lines made by transformation of human B cells with EBV constitutively produce FasL protein.

We next sought to determine the cellular localization of FasL in LCL by flow cytometry. To this end, we stained the surface of LCL cells with anti-FasL antibody or an appropriate isotype control antibody. Somewhat surprisingly, we found little or no detectable FasL on the surface of all LCL tested (Figure 1B). After fixation and permeabilization, however, we were able to detect intracellular FasL in all LCL tested (Figure 1B). It therefore appears that while LCL constitutively produce FasL protein, very little FasL is present on the cell surface under normal culture conditions.

### LCL secrete exosomes containing FasL and MHC class II

**Figure 2:**
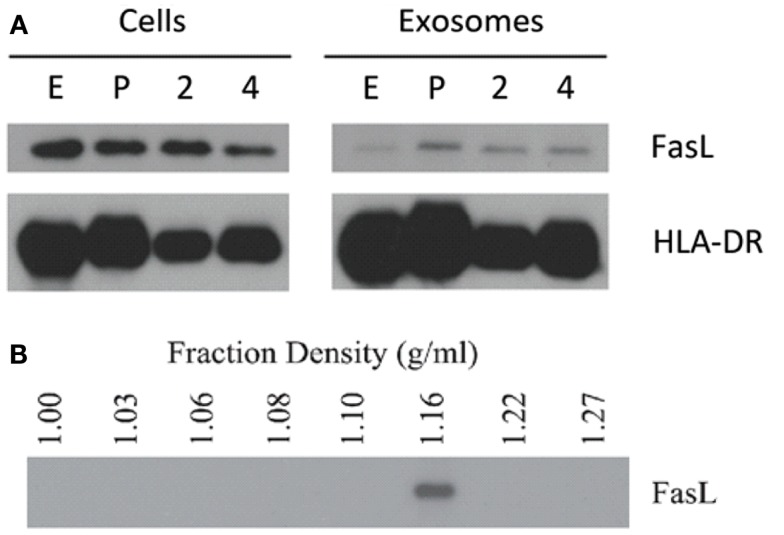
**LCL secrete exosomes containing FasL and MHC class II**. **(A)** Cell lysates and spontaneously secreted exosomes were collected from four LCL (lines E, P, 2, and 4) and probed for the presence of FasL and HLA-DR by immunoblot. **(B)** Exosomes from a representative LCL were floated onto a discontinuous density gradient of iodixanol solution and spun for 1 h at 100,000 × *g*. Individual layers were removed and diluted with PBS, followed by an overnight centrifugation at 100,000 × *g*. The resulting pellets were lysed with SDS buffer and probed for FasL by immunoblot.

Lymphoblastoid cell lines are known to spontaneously secrete exosomes, but there are no reports of LCL-derived exosomes containing FasL (2). Additionally, the secretory lysosome is the default destination for FasL in cells, which possess this compartment (24). We therefore hypothesized that as LCL express robust amounts of FasL, this FasL is likely to be sorted to the secretory lysosome and secreted on exosomes. To test this hypothesis, we collected supernatants from several independent LCL and isolated exosomes from these supernatants using ultracentrifugation. Briefly, cells and large debris were removed from supernatants by centrifugation at 500 × *g* and 10,000 × *g*, respectively. To pellet exosomes, the cleared supernatants were spun at 100,000 × *g* for 1–4 h. The resulting exosome pellets were lysed with SDS buffer and probed for expression of FasL and HLA-DR by immunoblot. FasL was detectable in the exosome fraction from all LCL tested (Figure 2A). We also confirmed that MHCII molecules were present in LCL-derived exosomes, as we found abundant HLA-DR in the exosome pellets as well (Figure 2A).

The pellet obtained by centrifuging supernatants at 100,000 × *g* can potentially be contaminated with large soluble complexes or other types of cell debris. It has been reported that exosomes have a characteristic density distinct from other membrane fragments or microparticles (2). We therefore centrifuged re-suspended LCL-derived exosomes through a discontinuous density gradient made by serial dilution of iodixanol in PBS. After spinning for 1 h at 100,000 × *g*, each layer was harvested and diluted in PBS, and centrifuged again at 100,000 × *g* overnight. The resulting pellets were then lysed in SDS buffer and probed for the presence of FasL by immunoblot as in Figure 1A. FasL protein was detected only in the fraction with a density of 1.16 g/mL (Figure 2B), a density indicative of exosomes (2). Taken together, these data demonstrate that exosomes containing FasL are constitutively secreted by LCL.

### Double-positive FasL^+^MHCII^+^ exosomes are present among LCL-derived exosomes

**Figure 3:**
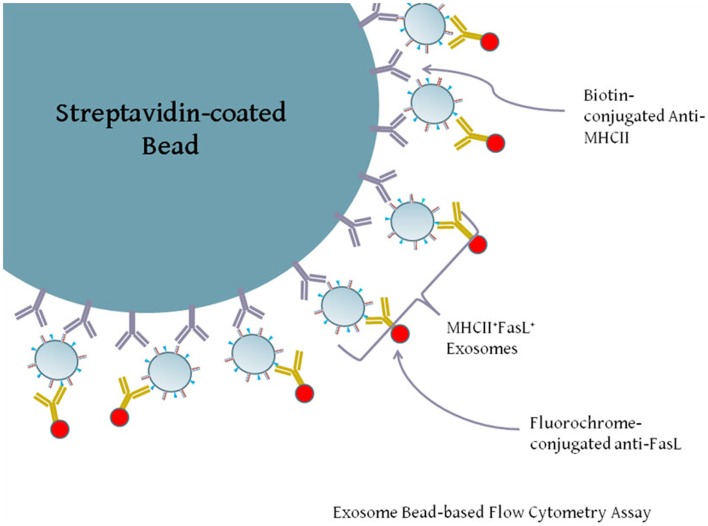
**Diagram of exosome-bead capture experiments**. Exosomes are too small to be accurately detected by standard flow cytometry, and so must be linked in aggregate to larger beads for flow cytometric analysis. Polystyrene beads coated with streptavidin were incubated with biotin-conjugated anti-HLA-DR or an isotype control antibody. Beads were then washed and incubated with gentle agitation for several hours with LCL-derived exosomes. Beads were washed and stained with PE-conjugated anti-FasL or an appropriate isotype antibody. Positive staining for FasL indicates the presence of MHCII^+^FasL^+^ exosomes.

Our previous experiments demonstrated that both FasL and MHCII protein were present in exosomes secreted by LCL. While both proteins are present on exosomes, it is not clear from the previous data whether individual exosomes possess both FasL and MHCII (FasL^+^MHCII^+^), or whether FasL and MHCII are present on distinct subsets of exosomes. Exosomes are too small to be accurately detected by standard flow cytometry, and so must be linked in aggregate to larger beads for flow cytometric analysis. We therefore developed an assay to capture exosomes on an antibody-coated bead and stain the captured exosomes with fluorescently conjugated antibodies (Figure 3). Polystyrene beads coated with streptavidin were incubated with a biotinylated antibody and washed several times. Antibody-coated beads were then incubated with exosomes, and after washing away excess exosomes, those bound to the beads were stained with anti-FasL antibody (Figure 3).

**Figure 4:**
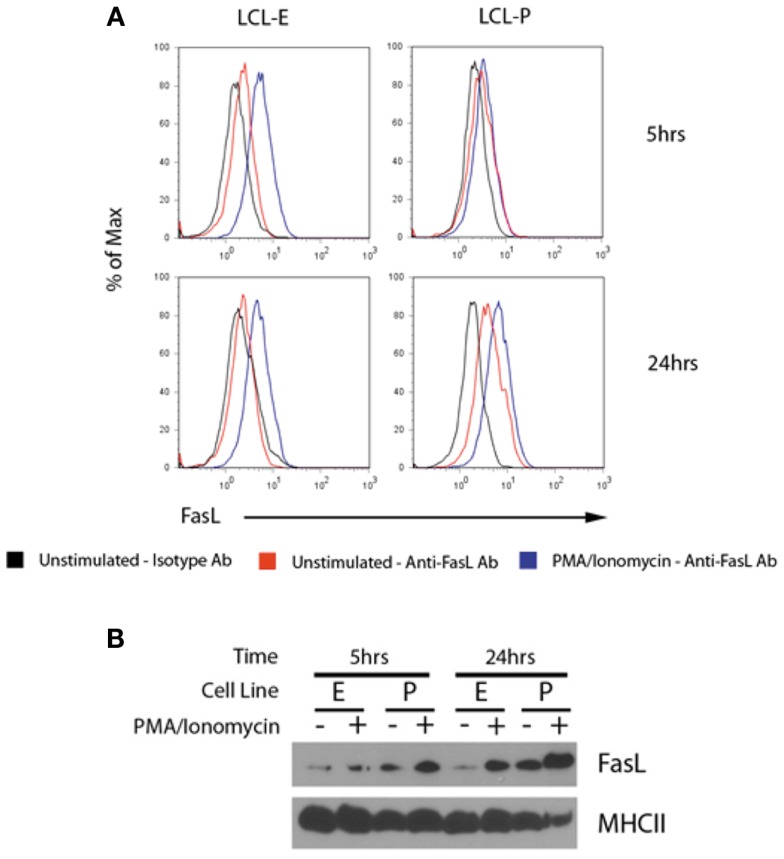
**LCL secrete MHCII^+^FasL^+^ exosomes**. **(A)** Two LCL (designated “LCL-E” and “LCL-P”) were placed into fresh exosome-free media in the presence or absence of PMA/ionomycin. After 5 h (top panels) or 24 h (bottom panels), exosomes were isolated from culture supernatants as described in the section “Materials and Methods.” Purified exosomes were then incubated with anti-HLA-DR-coated beads as described in Figure 3. Beads were washed and stained with either anti-FasL or an isotype control antibody. Beads were coated with an appropriate isotype control antibody as the capture antibody did not display any FasL staining (data not shown). **(B)** Cells from above experiment were harvested, lysed, and probed for FasL and MHCII (HLA-DR) by immunoblot.

To test for the co-localization of MHCII and FasL into the same exosomes, we harvested exosomes from unstimulated and PMA/ionomycin-stimulated LCL culture supernatants and concentrated them by centrifugation. Exosomes were then incubated with beads coated with anti-MHCII antibody, and stained with anti-FasL or isotype control antibodies. As can be seen in Figure 4A, exosomes bound to anti-MHCII-coated beads stained positive for the presence of FasL, demonstrating that FasL and MHCII are found on the same LCL-derived exosomes. Stimulation with PMA/ionomycin increased the amount of FasL detected on MHCII^+^ exosomes in both LCL tested (Figure 4A). Cells from these experiments were also lysed and probed for FasL, and both cell lines had increased cellular FasL protein levels in response to PMA/ionomycin stimulation (Figure 4B). Therefore, LCL secrete MHCII^+^FasL^+^ exosomes, and both the production of FasL protein and the release of MHCII^+^FasL^+^ exosomes can be increased by stimulation with PMA/ionomycin.

### LCL-derived exosomes can induce apoptosis in autologous CD4^+^ T cells

**Figure 5:**
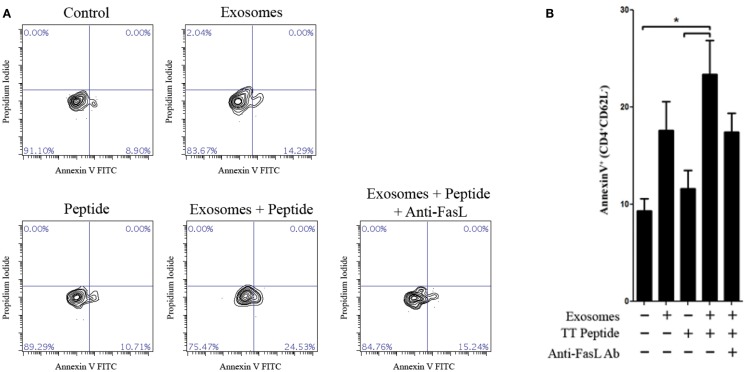
**Antigen-specific killing with TT peptide**. Peripheral blood mononuclear cells were harvested from a repeat donor from whom we had previously generated an LCL. This donor had received a scheduled vaccination against tetanus <1 month prior to the experiment shown. PBMCs were cultured in the presence of an immunodominant peptide of the tetanus toxoid for 12 days. CD4^+^ T cells were then isolated by negative selection and cultured overnight with or without autologous LCL-derived exosomes (156 μg/mL) and in the presence or absence of the tetanus toxoid peptide. Neutralizing anti-human FasL antibodies (10 μg/mL) were added to some wells to block interactions between exosome FasL and Fas on the target cells. Apoptosis in activated CD4^+^CD62L^neg^ T cells was assessed by annexin-V/propidium iodide staining. Two additional experiments with the same donor cells showed the similar trends but were less robust, presumably due to the loss of tetanus-specific T cells *in vivo* as the time after vaccination increased. **(A)** Representative contour plots of annexin-V and propidium iodide staining among CD4^+^CD62L^low^-gated T cells. **(B)** Frequency of annexin-V^+^ cells (mean ± SEM of triplicate samples) among CD4^+^CD62L^low^ T cells. *p < 0.05.

Since LCL-derived exosomes contained a measurable amount of MHCII^+^FasL^+^ exosomes, we hypothesized that activated CD4^+^ T cells would be susceptible to exosome-induced apoptosis. To test this hypothesis, we obtained PBMCs from a healthy donor who had been recently immunized against tetanus and from whom we had previously made an LCL and could collect autologous exosomes. T cells specific for a nominal antigen are rare among peripheral CD4^+^ T cells, and therefore detecting the capacity of LCL-derived exosomes to induce peptide antigen-specific apoptosis requires prior activation to enrich for peptide-specific T cells. PBMCs from this donor were cultured with the immunodominant peptide of TT to enrich the CD4^+^ T cell pool for cells specific to this antigen. After 12 days in culture, CD4^+^ T cells were isolated by negative selection. To demonstrate the antigen-specificity of exosome-mediated T cell apoptosis, the same TT peptide was introduced in excess to a portion of LCL-derived exosomes to displace peptides already present on the exosome MHC class II molecule. CD4^+^ T cells were then incubated overnight with autologous LCL-derived exosomes in the presence or absence of the TT peptide. Apoptosis was assessed by annexin-V/propidium iodide staining among activated T cells (CD4^+^CD62L^neg^). LCL-derived exosomes in the presence of TT peptide induced significant levels of apoptosis in CD4^+^ CD62L^neg^ T cells (Figure 5). While exosomes that were not loaded with TT peptide also induced apoptosis above baseline, this difference did not reach statistical significance (Figure 5). The addition of a neutralizing anti-FasL antibody to culture with exosomes and TT peptide appeared to inhibit exosome-induced apoptosis, but this difference also did not reach statistical significance (Figure 5). Taken together, these data suggest that LCL-derived exosomes can induce apoptosis in activated T cells that is antigen-dependent and may be at least partially mediated by FasL.

**Figure 6:**
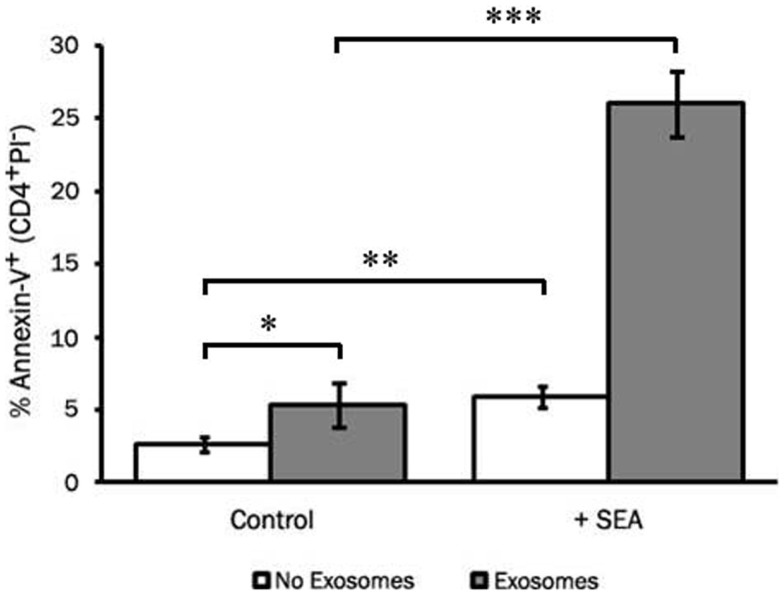
**LCL-derived exosomes can induce apoptosis in autologous CD4^+^ T cells**. CD4^+^ T cells were harvested from a repeat donor from whom we had previously generated an LCL. CD4^+^ T cells were incubated with exosomes (127 μg/mL) in the presence or absence of the super-antigen, staphylococcal enterotoxin A (SEA). After 6 days in culture, PBMCs were harvested and apoptosis was assessed in CD4^+^ T cells by annexin-V/propidium iodide staining. Data are representative of three independent experiments using exosomes from this donor. Similar results were obtained using exosomes from a different LCL donor (data not shown). *p < 0.05, **p < 0.01,***p < 0.001.

To assess the ability of LCL-derived exosomes to induce apoptosis of T cells that had not previously been exposed to antigen, we employed the super-antigen, staphylococcal enterotoxin A (SEA), to facilitate interactions between MHC class II and the T cell receptor. LCL-derived exosomes were isolated from unstimulated LCL culture supernatant as described above and cultured with fresh CD4^+^ T cells in the presence or absence of SEA. After 6 days in culture, we assessed apoptosis in CD4^+^ T cells by annexin-V/propidium iodide staining. In the absence of SEA, LCL-derived exosomes produced a modest increase in apoptosis in CD4^+^ T cells (Figure 6). In contrast, in the presence of SEA, exosomes induced significant levels of apoptosis in CD4^+^ T cells (Figure 6). Similar results were obtained using an LCL and CD4^+^ T cells from a second independent donor (data not shown). Taken together, these results suggest that LCL-derived exosomes can mediate antigen-specific killing of CD4^+^ T cells.

## Discussion

Exosome-mediated immunotherapy for the treatment of inflammatory disorders is an intriguing concept because MHCII^+^FasL^+^ exosomes have demonstrated permanent and precisely focused suppression of antigen-specific immune responses in mouse models (4, 5). For the treatment of human inflammatory conditions, exosomes may represent a safer alternative to regulatory cells for immunotherapy because the phenotype of exosomes is expected to be static, whereas regulatory cells can potentially differentiate into effector cells after transfer (7). Although this technique has promise, a reliable method for producing donor-derived immunosuppressive exosomes is required before this therapeutic strategy can be developed as a strategy to induce and maintain tolerance after transplantation. We show here that transformation of human B cells with EBV results in robust expression of FasL, and that all LCL tested in this study also produced MHCII^+^FasL^+^ exosomes. Generating LCL from peripheral blood B cells is widely practiced and a relatively simple process, requiring only minimal laboratory labor and reagents. The resulting transformed B cells can be grown to high concentrations and stored over long periods of time. Therefore, LCL represent a potentially reliable source of immunosuppressive exosomes from any donor that could be therapeutically useful in humans.

B cells expressing FasL are relatively infrequent under most conditions, and we therefore hypothesized at the outset of this project that FasL expression among human B cell-derived tumor lines would be rare as well. While FasL expression was indeed infrequent among primary B cells and cell lines derived from other types of B cell cancers, we found that FasL protein was present in cell lysates from all LCL tested in this study. This result was somewhat surprising as LCL are reportedly susceptible to FasL-induced apoptosis, and LCL have been used extensively as APC for activating T cells (25–28). These conflicting results can be explained in part by the fact that unlike endogenous FasL^+^ B cells in mice, FasL protein is undetectable on the surface of LCL. Therefore, although LCL produce FasL, this intracellular sequestration makes it unavailable for inducing apoptosis in target cells unless transported to the cell surface or released. In the present study, we demonstrate that PMA/ionomycin stimulation triggers increased production of FasL protein by LCL, as well as the release of FasL^+^MHCII^+^ exosomes. These data suggest that antigen receptor- and calcium-dependent signaling pathways are involved in the regulation of FasL^+^ exosome transport. Other studies have demonstrated that ligation of ICAM-1, B7-H1, or B7-H4 on cultured LCL leads to activation of reactive oxygen species, which in turn cause the translocation of FasL to the cell surface and induction of LCL apoptosis, however the release of FasL^+^ exosomes was not measured (29–31).

The high frequency of FasL expression and exosome release among LCL suggests that FasL may be an important component of natural EBV infection. LCL are generated experimentally by infection with the B95-8 strain of EBV, which is a replication incompetent form of the native γ-1 herpes virus that has oncogenic potential in humans (32). The natural virus persists in most infected individuals in latently infected circulating memory B cells (33, 34). Greater than 90% of adults have been infected with EBV, and although clinical manifestations of infection are generally rare, the transforming properties of the virus can lead to B cell-derived malignancies such as Burkitt’s and Hodgkin lymphomas (35, 36). LCL generated by infection with EBV maintain a latent viral growth program, expressing at least eight proteins from the viral genome (35). Among these proteins is latent membrane protein 1 (LMP1), a functional mimic of CD40 (37). Signaling of LMP1 differs from that mediated by CD40, as LMP1 signaling is constitutive rather than ligand-dependent. Therefore, LCL are essentially in a state of constant CD40 stimulation. We previously observed that mouse B cells stimulated *in vitro* with CD40L and IL-5 express higher levels of FasL (38), and therefore the CD40-mimicry of LMP1 might potentially explain the constitutive production of FasL in LCL. Additionally, stimulation with CD40L has been reported to induce FasL expression in other types of cells (39–41).

The expression of FasL by LCL and their production of FasL^+^/MHCII^+^ exosomes may have important implications in clinical management of EBV infections. While most people are first exposed to EBV in infancy, those infected later in life can develop acute infectious mononucleosis (AIM) (42). At the height of acute infection, T cells are susceptible to Fas-mediated apoptosis, and *in vitro* infection of PBMCs with EBV leads to elevated levels of FasL on the surface of B cells (43). Inducing surface expression of FasL in B cells may therefore be a means of immune evasion employed by EBV during the lytic cycle (44). Importantly, FasL localization appears to differ between the lytic and latent cycles, as FasL in LCL is intracellular whereas FasL can be found on the surface of B cells during acute infection (43). As the virus transitions into a latent state and settles into homeostasis with the host immune system, infected B cells may maintain FasL production but cease transporting it to the cell surface. This may represent a natural mechanism by which LCL can persist without causing significant tissue damage while maintaining the ability to ward off elimination by virus-specific T lymphocytes.

We demonstrate here that LCL constitutively produce MHCII^+^FasL^+^ exosomes with apoptosis-inducing activity against CD4^+^ T cells. Although FasL can be detected in LCL-derived exosomes under normal conditions, stimulation with PMA/ionomycin increased both the amount of MHCII^+^FasL^+^ exosomes secreted and FasL production in LCL. However, while FasL is abundant in LCL cell lysates, it is relatively difficult to detect in exosomes, even after stimulation. In contrast, the expression of MHC class II was very abundant in exosomes in comparison to FasL. Based on these findings, we hypothesize that LCL may produce a mixture of MHC class II^+^ exosomes that are either FasL^+^ or FasL^−^. It has proven difficult to separate these potential LCL-derived exosome subsets using current techniques (45, 46). Some limitations were created because anti-human or mouse FasL antibodies have been unable to capture exosomes (data not shown), and the beads used for exosome capture have multiple binding sites that can potentially capture several different exosomes simultaneously. These are important limitations to the current study because there is likely to be a balance of stimulation with and without concomitant T cell apoptosis in our experimental model system. The balance of these two forces may explain some seemingly contradictory results that have been published regarding the immune modulatory properties of adoptively transferred exosomes *in vivo* (6, 47). Notably, most studies showing stimulation of immune responses by adoptive transfer of exosomes in mice have not included an assessment of exosome FasL expression or apoptosis. Until better methods of FasL^+^ exosome capture or separation can be developed, it will remain difficult to analyze their immunotherapeutic potential in complex animal models such as allotransplantation.

Despite the likelihood of contamination by FasL^−^ exosomes, the exosomes were able to induce apoptosis in approximately 25% of the T_H_ cells in the cultures when super-antigen or an antigenic peptide with which the T_H_ cells had been previously activated were present. The partial blockade of killing when anti-human FasL antibody was added to the culture indicates that there may be additional mechanisms of cell death occurring. This is not surprising given the heterogeneity of the exosome preparation and the potential for other death ligands such as TRAIL or TNF to be expressed on exosomes (40, 48). It will be interesting to determine how parameters such as dose, timing, and efficiency of eliminating allo-specific T cells *in vitro* and *in vivo* will be affected by improvements in purification of FasL^+^ exosomes.

There remain many important considerations to address before FasL^+^ exosomes can be used in clinical trials. A top priority will be to develop the methods of selectively and efficiently separating FasL^+^MHCII^+^ exosomes from FasL^−^ exosomes. Our data show that FasL^+^ exosomes were highly enriched at a specific density of 1.16 g/mL. This fraction also contained MHCII molecules (data not shown), however, several other density fractions also had MHCII at similar levels without FasL, suggesting that FasL^+^ exosomes are only a small component of exosomes released by LCL. Density gradient centrifugation may be a useful method for enriching FasL^+^ exosomes, but it may prove more efficient to isolate these exosomes by immunoaffinity capture techniques (49). Once reliable methods have been developed to purify FasL^+^ exosomes, it should be feasible to demonstrate their antigen-specific immune suppressive properties using *in vitro* mixed lymphocyte reactions and *in vivo* adoptive transfer models in mice.

Another important caveat to consider is the effect of such a therapy on the ability of the recipient to control latent EBV infections, as >90% of patients may harbor endogenous virus. While CD8^+^ T cells among PBMCs are relatively resistant to FasL-mediated apoptosis, it may still be desirable to reduce MHC class I levels in LCL-derived exosomes to prevent them from interacting with CD8^+^ T cells after transfer (50). This could be accomplished by introducing engineered gene-specific nucleases that can target genes important for MHC class I expression in the recombinant EBV genome, such as transporter associated with antigen processing (TAP) or β_2_ microglobulin (51, 52). Such a strategy would expectedly reduce the amount of MHC class I on exosomes, and therefore reduce the likelihood that LCL-derived exosomes would deplete EBV-specific CD8^+^ T cells in LCL-exosome recipients. CD4^+^ T cell immunity also appears to be crucial for controlling the latent EBV infection (53). To overcome the potential loss of virus-specific CD4^+^ T cells, it may be necessary to vaccinate recipients of LCL-derived FasL^+^ exosomes post-transplantation to reestablish immunity against EBV.

Despite the concerns listed above, there are many positive aspects to using EBV-transformed B cells as a source of tolerogenic exosomes. Generating LCL with B95-8 EBV has proven to be a simple, reliable, and safe method that has already been used to produce thousands of lines that could serve as a source of FasL^+^ exosomes. Huge repositories of LCL exist containing many lines that have been genotyped for MHC class I and class II expression and the high frequency of FasL expression by LCL also makes it feasible to generate *de novo* LCL from people on organ donor registries with exact MHC representation. EBV transformation results in a homogeneous culture of immortalized B cells that grow rapidly, can be maintained at high cell densities, and does not require cell sorting to remove contaminating cell populations. Exosomes are reportedly stable in phenotype and can be stored over long periods of time without significant loss of function (54). The genome of EBV is maintained in proliferating LCL as a large episome (~167 kb), and techniques for engineering recombinant EBV are well-established (55, 56). The large size of the viral genome will allow for the addition of transgenes to the virus, such as a segment containing the FasL gene under a strong, ubiquitous promoter to ensure robust production of FasL. Additionally, recombinant EBV can be produced containing the coding sequence for tissue-specific alloantigens fused to a lysosomal sorting sequence. Proteins containing this sequence are actively sorted to the secretory lysosome where they are processed and presented on MHCII molecules (57). Thus, MHCII^+^FasL^+^ exosomes produced by such a LCL could be engineered to present various epitopes of the alloantigen. The resulting exosomes could be harvested under sterile conditions by centrifugation, affinity, or filtration, and either frozen for future use or administered directly to the patient.

The utility of LCL-derived FasL^+^ exosomes to tolerize allograft recipients remains to be determined. Other potential uses of these immune suppressive exosomes could include treatments for T cell-mediated allergies and autoimmune diseases, since the goal of eliminating antigen-specific T cells is similar in these conditions. Alternatively, the development of effective tumor vaccines using exosomes, which is currently being intensely studied, may be dependent on the removal or suppression of FasL^+^ exosomes from the preparation. An important consideration is that exosomes have the ability to travel relatively far away from the cells that produced them, and yet perform many of the same functions that have been previously attributed to direct cell–cell contact. Much controversy has existed over the functions of soluble forms of FasL, yet until recently there was no distinction made between truly soluble FasL and vesicular FasL. The recent developments in the field of exosome research should cause a reassessment of what we think is known about cellular interactions in the immune system and consequently to our approaches toward immunotherapy.

## Conflict of Interest Statement

The authors declare that the research was conducted in the absence of any commercial or financial relationships that could be construed as a potential conflict of interest.
